# Assessment of the electrical penetration of cell membranes using four-frequency impedance cytometry

**DOI:** 10.1038/s41378-022-00405-y

**Published:** 2022-06-24

**Authors:** Tao Tang, Xun Liu, Yapeng Yuan, Tianlong Zhang, Ryota Kiya, Yang Yang, Kengo Suzuki, Yo Tanaka, Ming Li, Yoichiroh Hosokawa, Yaxiaer Yalikun

**Affiliations:** 1 Division of Materials Science, Nara Institute of Science and Technology, 8916-5 Takayama-cho, Ikoma, Nara, 630-0192 Japan; 2 Center for Biosystems Dynamics Research (BDR), RIKEN, 1-3 Yamadaoka, Suita, Osaka, 565-0871 Japan; 3 School of Engineering, Macquarie University, Sydney, 2109 NSW Australia; 4 Institute of Deep-Sea Science and Engineering, Chinese Academy of Sciences, Sanya, Hainan 572000 P. R. China; 5 Euglena Co. Ltd., Tokyo, 108-0014 Japan

**Keywords:** Electrical and electronic engineering, Chemistry

## Abstract

The electrical penetration of the cell membrane is vital for determining the cell interior via impedance cytometry. Herein, we propose a method for determining the conductivity of the cell membrane through the tilting levels of impedance pulses. When electrical penetration occurs, a high-frequency current freely passes through the cell membrane; thus, the intracellular distribution can directly act on the high-frequency impedance pulses. Numerical simulation shows that an uneven intracellular component distribution can affect the tilting levels of impedance pulses, and the tilting levels start increasing when the cell membrane is electrically penetrated. Experimental evidence shows that higher detection frequencies (>7 MHz) lead to a wider distribution of the tilting levels of impedance pulses when measuring cell populations with four-frequency impedance cytometry. This finding allows us to determine that a detection frequency of 7 MHz is able to pass through the membrane of *Euglena gracilis* (*E. gracilis*) cells. Additionally, we provide a possible application of four-frequency impedance cytometry in the biomass monitoring of single *E. gracilis* cells. High-frequency impedance (≥7 MHz) can be applied to monitor these biomass changes, and low-frequency impedance (<7 MHz) can be applied to track the corresponding biovolume changes. Overall, this work demonstrates an easy determination method for the electrical penetration of the cell membrane, and the proposed platform is applicable for the multiparameter assessment of the cell state during cultivation.

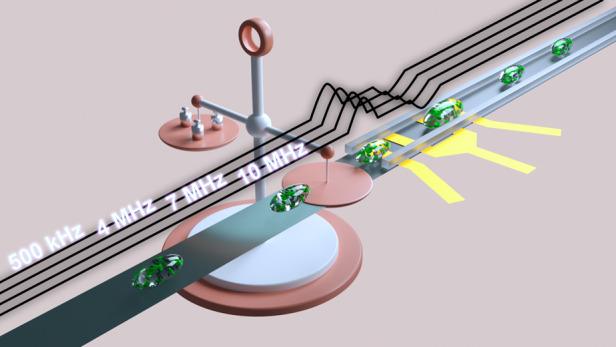

## Introduction

The biomass assessment of single cells plays a vital role in many areas, including the analysis of the cell state^1^ and cell growth mechanism^2^, as well as environmental and energy issues^3,4^. To date, several techniques, including live-cell imaging^5^, Raman flow cytometry^6^, and chemical probes^7^, have been successfully applied for the high-throughput assessment of intracellular biomass in single cells. However, most of these optical-based approaches are time-consuming and labor intensive, and the tight requirements for maintaining and calibrating beam-focusing points limits their robustness and portability. In this work, we proposed a more effective and convenient method for characterizing biomass through the magnitudes of high-frequency impedance signals.

As an alternative, impedance cytometry has been demonstrated to be applicable for single-cell characterization in a label-free and cost-effective manner^8^. To date, impedance cytometry has been successfully employed to analyze the morphology^9^, stiffness^10^, and states^11^ of single cells. The magnitude and morphology of impedance pulses have been shown to be dependent on the volume^12^ and shape^13^ of single cells, respectively. In addition, research has found that high-frequency impedance detection is applicable for characterizing membrane properties^14,15^. For example, the conductivity of the cell membrane increases with increasing detection frequency above 1 MHz^14^, and the membrane conductivity is related to the cell viability. Sui et al.^16^ and Zhong et al.^17^ have shown that a detection frequency of 5–8 MHz is sufficient to allow current to pass through the membranes of the living cells of mammals. This conclusion is drawn from the differences in membrane conductivity between inactivated and living cells. Without benchmarking against inactivated cells, there are few reports of applicable methods for directly determining whether the membrane is conductive.

When measuring intracellular biomass with impedance cytometry, it is necessary to determine the detection frequency that can penetrate the cell membrane. Our solution is to quantify the tilt level of impedance pulses as a tilt index^13^ and then assess the conductivity of the cell membrane through the tilt index of the cell population at different detection frequencies. Based on our previous work, the intracellular component distribution was found to affect the tilting level of high-frequency impedance pulses (6 MHz)^18,19^ because a high-frequency current can propagate inside single cells between nonconductive intracellular components. In contrast, a low-frequency current (500 kHz) cannot penetrate the cell membrane, and it mainly propagates around the cell^18,19^. This feature facilitates a novel method for determining the detection frequency of the cell interior and exterior based on the tilt index of impedance pulses. Cell interiors are more heterogeneous than their morphologies in a population. When the detection frequency is high enough to penetrate the cell membrane, the tilt index of the impedance pulses for a cell population will be more varied. To our knowledge, this is the first time that the tilting level of impedance pulses has been used to determine the detection frequency of the cell interior.

**Fig. 1:**
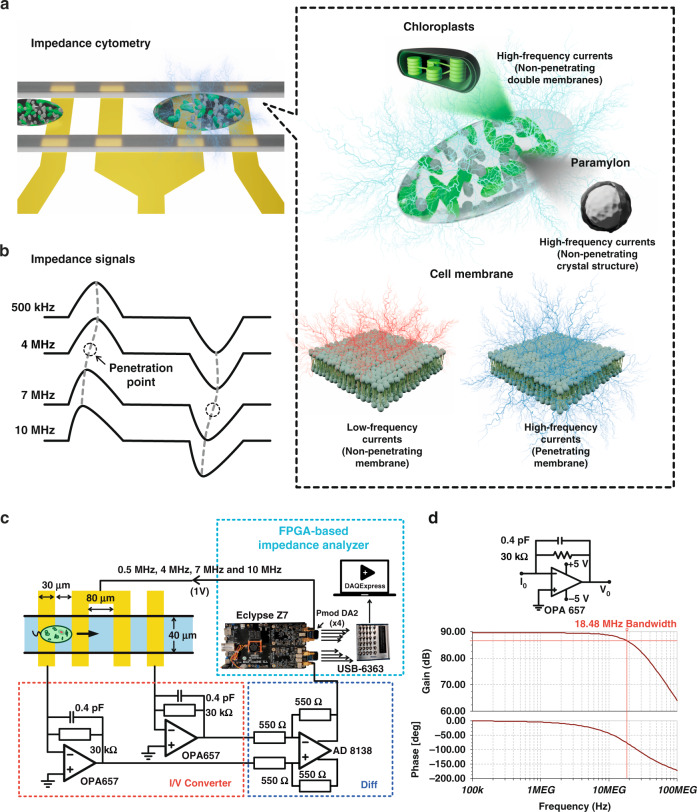
Schematics of four-frequency impedance cytometry. **a** Microfluidic impedance cytometry for the detection of *E. gracilis* cells and some important structures of single *E. gracilis* cells. **b** Impedance signals at four distinct frequencies (i.e., 500 kHz, 4 MHz, 7 MHz, and 10 MHz) and the effect of intracellular component distribution on the morphology of high-frequency impedance signals. **c** Impedance analyzer and (**d**) Frequency response of the self-developed transimpedance amplifier

In this work, four-frequency impedance cytometry was employed to analyze the conductivity of single *Euglena gracilis* (*E. gracilis*) cells, as shown in Fig. 1a. First, impedance detection at different frequencies of single *E. gracilis* cells was used to determine at which detection frequency the current could pass through the cell membrane. When a high-frequency electrical field penetrates the cell membrane, the uneven intracellular distribution tilts the impedance pulses to the left or right (see Fig. 1b), which is a phenomenon that has been verified in simulations and experiments. Additionally, the proposed four-frequency impedance cytometry technique (i.e., 500 kHz, 4 MHz, 7 MHz, and 10 MHz) was applied to monitor the biomass of single *E. gracilis* cells from four-day cultures under various conditions based on the ability of high-frequency impedance to detect intracellular biomass. The electrical scanning of *E. gracilis* cells internally and externally clearly showed cellular responses to different cultivation media with organic sources or inorganic ions, in which cells exhibited significant differences in multiplication, volume, and opacity. The volume of single cells was monitored via low-frequency impedance magnitudes, and their biomass changes were tracked by high-frequency impedance magnitudes. The impedance detection system was built on a field-programmable gate array (FPGA) board (see Fig. 1c) with a homemade transimpedance amplifier (see Fig. 1d)^20^. We envision that the tilt index can facilitate an alternative method for determining the frequency of electrical penetration for cell membranes. In addition, the proposed impedance-based platform can be adopted to evaluate cellular states and biomass, which is critical in practical applications involving continuous cell cultures^21,22^.

## Results

### Tilt index

**Fig. 2:**
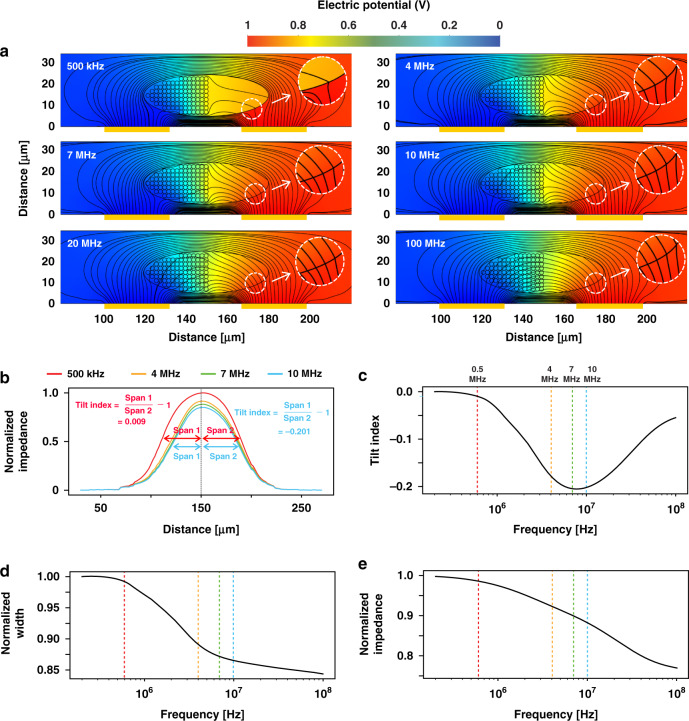
Simulation analysis for the penetration frequency. **a** Electric potential distribution at six different frequencies, including 500 kHz, 4 MHz, 7 MHz, 10 MHz, 20 MHz, and 100 MHz. The density of the black streamlines indicates the current density (A m^−2^). **b** Simulated impedance pulses at the four detection frequencies used in this work. **c**–**e** Three simulated spectra of frequencies vs. (**c**) tilt index, (**d**) normalized width, and (**e**) normalized impedance of the impedance pulses. Note that the impedance magnitude and the width of impedance pulses were normalized based on the low-frequency impedance metrics

To determine the penetration frequency of single cells, we performed 2D numerical simulation via the AC/DC Module of COMSOL 5.6 Multiphysics software (COMSOL Inc., Burlington, MA, USA). Herein, *E. gracilis* cells were simulated as single-shell ellipses (radius: 30 μm long-axis, 10 μm short-axis), and the cell membrane (10 nm) was modeled using contact impedance approximation^23^. Intracellular components were simplified as 2D circles (membrane thickness of 20 nm and diameter of 1 μm) and were closely placed in the left interior of the cell^18,19^, as shown in Fig. 2a. Other parameters of cells^18^ used in the simulation are listed in Table S1 in the Supplementary information.

In impedance detection, the conductivity of the cell membrane is frequency dependent, and as shown in Fig. 2a, the current density inside the cell gradually increases with increasing detection frequency. The seamless passage of high-frequency current through the cell membrane shows the applicability of high-frequency impedance detection for characterizing intracellular components. In the simulation, the increasing current density inside the cell indicates the strengthening capability of the high-frequency current to pass through the cell membrane as the detection frequency increases.

Figure 2b shows the impedance pulses induced the same cell model at four detection frequencies. As the detection frequency increases, the resistance of the cell model against current propagation decreases, resulting in smaller magnitudes of the impedance pulses. In addition, the impedance pulses corresponding to cells with symmetric shapes at a low detection frequency (500 kHz) are symmetric. In contrast, the right-hollow cell interior is responsible for inducing asymmetrical impedance pulses at high detection frequencies. The right half of the impedance pulses lasts longer than the left half. This phenomenon can be quantified by the tilt index, which is defined as the ratio of the time spans on either side of the impedance pulse minus one (see Fig. 2b). Specifically, the tilt index is 0.009 for symmetric impedance pulses at 500 kHz, while it is −0.201 for asymmetric impedance pulses at 10 MHz. More detailed information about the tilt index is provided in Fig. S1 in the supplementary information.

Figure 2c shows the dependency of the tilt index on the detection frequency from 100 kHz to 100 MHz. All tilt indices are benchmarked against the value (zero) at a low detection frequency of 100 kHz. An increasing tilt index indicates the increasing impact of the intracellular component distribution on the tilting level of the impedance pulses. As the detection frequency increases, the interior structure of the right-side of the entire cell gradually tilts the impedance pulses to the right. At approximately 10 MHz, the tilt index reaches an extreme value, and after that, the intracellular components start being electrically penetrated (see Fig. 2a). In comparison, the width and magnitude of impedance pulses (see Fig. 2d, e) contain little information about the intracellular component distribution. Both values decrease with increasing detection frequency because of the decreasing resistance of the cell membrane, as has been previously demonstrated in various studies^8,24^.

### Electrical penetration

**Fig. 3:**
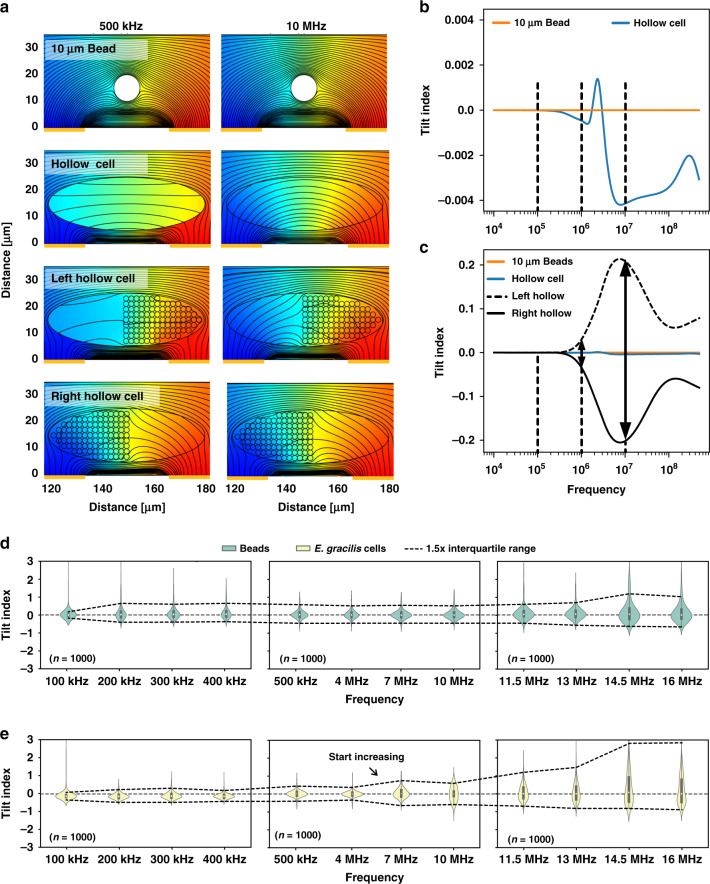
Simulation and experimental analysis of the electrical penetration of the cell membrane. **a** Electric potential distribution analysis at two different frequencies, 500 kHz and 10 MHz, based on four types of simulation models, including 10 μm beads, hollow cells, left-hollow cells and right-hollow cells. **b** Frequency dependence of the tilt index induced by 10 μm beads and hollow cells. **c** Frequency dependence of the tilt index induced by all four types of simulation models. **d**, **e** Violin plots of the experimental results of the tilt index induced by (**d**) 10 μm polystyrene beads and (**e**) *E. gracilis* cells at 12 different detection frequencies, including 100 kHz, 200 kHz, 300 kHz, 400 kHz, 500 kHz, 4 MHz, 7 MHz, 10 MHz, 11.5 MHz, 13 MHz, 14.5 MHz and 16 MHz

Cell interiors are more heterogeneous than their morphologies in a population. Heterogeneous internal structures cause the tilt index to become increasingly decentralized when the electrical field starts penetrating the cell membrane. The simulation results of the frequency dependence of the tilt index measured using four different models, including 10 μm beads, hollow cells, left-hollow cells and right-hollow cells, are presented in Fig. 3a–c. At detection frequencies ranging from 100 kHz to 100 MHz, the current cannot penetrate the nonconductive beads. Thus, the impedance pulses of the beads are always symmetric in shape, and the tilt indices remain zero (see Fig. 3b). In contrast, the shape of the impedance pulses for hollow cells is asymmetric once the current penetrates the cell membrane. For example, at detection frequencies above approximately 100 kHz, the tilt index starts increasing from zero, and it reaches a maximum value at approximately 10 MHz. According to this phenomenon, it is possible that the propagation of current inside the cell could cause the asymmetric shape of the impedance pulses.

Additionally, when there are components inside the cell membrane, the tilting levels of impedance pulses are more noticeable than when the cell interior is hollow (see Fig. 3c). Thus, the tilt index caused by the cell membrane can be ignored for normal cells in practical detection since there are always intracellular components, such as organelles or macromolecules, within the cell membrane. In addition, the tilt index shows a dependency on the intracellular component distribution with increasing detection frequency. There was almost no difference in the values of the tilt index induced by the right-hollow or left-hollow cell model, except for the sign. The tilt index measured for a left-hollow cell model is always positive when the detection frequency is sufficient to penetrate the membrane, since the left-hollow structure causes the impedance pulses to tilt to the left. For right-hollow cells, the tilt indices are always negative. Before intracellular components are polarized by high-frequency electrical fields, the difference in tilt index induced by the left-hollow or right-hollow cell models gradually increases. In addition, this difference occurring indicates that the cell membrane is electrically penetrated.

Figure 3d, e illustrate the tilt indices induced by *E. gracilis* cells and 10 μm beads, respectively. Four-frequency impedance cytometry was used to measure single cells or particles at 12 different detection frequencies. Three independent measurements were made, whereby we applied the first set of detection frequencies within 500 kHz (i.e., 100 kHz, 200 kHz, 300 kHz, 400 kHz), the second set between 500 kHz and 10 MHz (i.e., 500 kHz, 4 MHz, 7 MHz, and 10 MHz), and the third set above 10 MHz (11.5 MHz, 13 MHz, 14.5 MHz, and 16 MHz). In the case of nonconductive polystyrene beads, their tilt index varied within a stable range when frequencies lower than 13 MHz were applied. After that, a higher detection frequency resulted in a more decentralized distribution of the tilt index. This may be because the detection frequency (>13 MHz) exceeds the upper limit of our impedance detection system. For the *E. gracilis* cells, the tilt index starts decentralizing from 7 MHz; thus, the electric penetration of the cell membrane occurs. In comparison with the tilt index of polystyrene beads, device influence can be excluded when frequencies are lower than 13 MHz.

By comparing Fig. 3c and e, we can conclude that the increasing decentralization of the tilt index is indicative of the electrical penetration of the cell membrane. In this work, a frequency of 7 MHz is sufficient to penetrate the cell membrane for intracellular component detection. Our previous findings also support this conclusion^18,19^.

### Organic nutrients and biomass accumulation

After determining the electrical penetration frequency of the cell membrane, we employed low-frequency impedance metrics (i.e., 500 kHz and 4 MHz) to track the volume changes in *E. gracilis* cells during photomixotrophic cultivation, as well as high-frequency impedance metrics (i.e., 7 MHz and 10 MHz) to monitor biomass accumulation. We cultured *E. gracilis* cells in Koren-Hutner (KH) medium for four days, and impedance signals were used to determine the biomass accumulation of *E. gracilis* cells grown photomixotrophically. The impedance detection of *E. gracilis* cells is shown in Movie S1 and Fig. S2 in the supplementary information. In this work, a maximum detection frequency of 10 MHz was used, which worked well within our system and is also commonly used for cell interior analysis^8^. The lowest detection frequency (500 kHz) was utilized in our previous work to characterize the volume and shape of single cells^18,19^. The two middle frequencies, namely 4 MHz and 7 MHz, were selected based on a 3 MHz spacing.

**Fig. 4:**
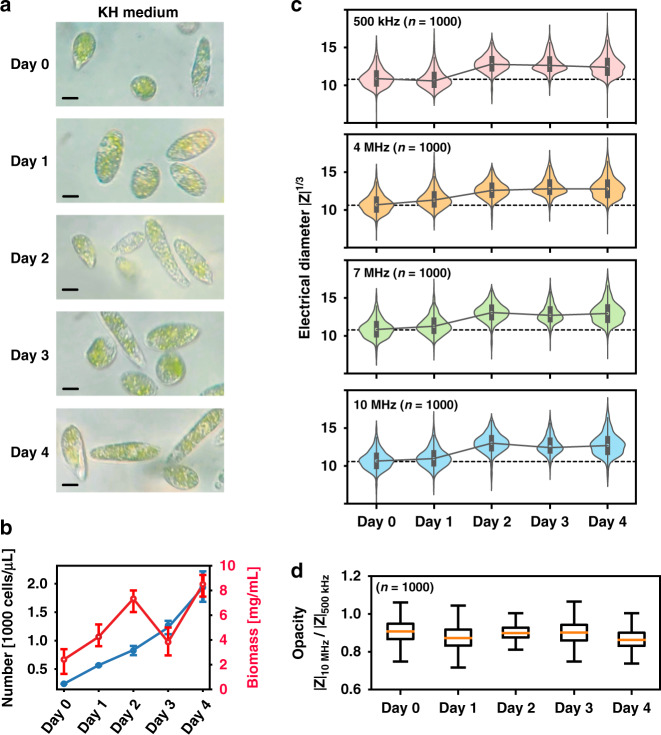
Cell cultivation in KH medium over four days. **a** Comparison of the volume of *E. gracilis* cells within four-day experiments. The scale bar indicates 10 μm. **b** Statistical analysis of the cell proliferation and biomass accumulation of *E. gracilis* cells. **c** Time course of changes in the electrical diameters of *E. gracilis* cells at four detection frequencies (500 kHz, 4 MHz, 7 MHz and 10 MHz). **d** Time course of changes in the electrical opacity of *E. gracilis* cells

*E. gracilis* cells can proliferate rapidly and accumulate paramylon in photomixotrophic cultivation by either photosynthesis or digesting organic carbon sources in the cultivation medium (KH medium). The *E. gracilis* cells cultivated in KH medium over four days are illustrated in Fig. 4a). As shown in Fig. 4b, the number of *E. gracilis* cells continued to increase over four days of cultivation, from approximately 241 cells/μL to 1936 cells/μL. Additionally, the biomass of *E. gracilis* cells increased rapidly from 2.4 mg/mL to 8.5 mg/mL. The sudden drop at Day 3 may be due to measurement error.

For the impedance characterization of single cells, shown in Fig. 4c, all dielectric properties of *E. gracilis* cells were calibrated using the dielectric properties of 10 μm beads. Over a four-day cultivation period, the electrical diameter of cells at 500 kHz increased from approximately 10.89–11.46. This is because the low-frequency impedance value depends on the cell volume: a rise in low-frequency electrical diameters indicates an increase in cell volume^8,18,19,24,25^. At the highest detection frequency (10 MHz), current can freely penetrate the cell membrane and propagate in the cytoplasm between intracellular components (i.e., paramylon and chloroplasts), allowing the high-frequency electrical diameter to be related to the intracellular nonconductive biomass of individual cells. Therefore, increases in both the low- and high-frequency diameters indicate that there were slight increases in the volume and biomass during the first 2 days.

In Fig. 4d, the electrical opacity of the cells (Days 1–4) is nearly identical to that of the precultures (Day 0) since the new cultivation conditions are the same as the preculture conditions. However, a rise in the high-frequency electrical diameter (i.e., 7–10 MHz) occurred on the first day, earlier than the increase in the low-frequency (i.e., 500 kHz) electrical diameter that occurred on the second day (see Fig. 4c). This may be because when the *E. gracilis* cells were transferred to a fresh medium, adequate organic nutrients and inorganic ions induced the generation of intracellular components prior to cell multiplication. In detail, the proliferation rate of *E. gracilis* cells can be accelerated by the ions Mg^2+^, Ca^2+^, Mn^2+^, Cu^2+^, Co^2+^, and Ni^2+^ in the medium^26^, and cell multiplication occurs slightly later than chloroplast multiplication. For *E. gracilis* cells, the number of chloroplasts in each cell is relatively stable, varying from 10 to 20^27^. When there are 60 or more chloroplasts per cell, cell multiplication usually occurs^28,29^. Thus, *E. gracilis* cells may have increasing intracellular biomass prior to their multiplication, which results in a slightly earlier increase in the high-frequency electrical diameters compared to that of the low-frequency electrical diameters.

### Inorganic ions and cell multiplication

**Fig. 5:**
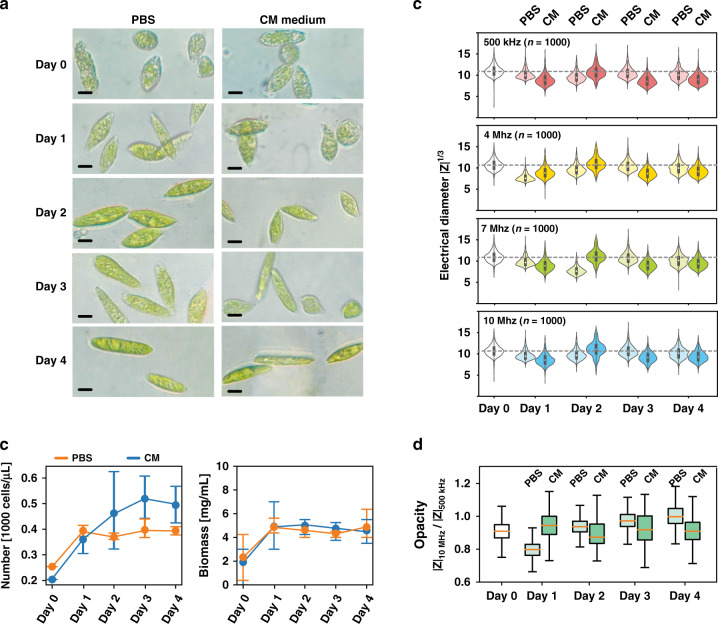
Cell cultivation in PBS solution and CM medium over four days. **a** Comparison of the volume of *E. gracilis* cells during four-day experiments. The scale bar indicates 10 μm. **b** Statistical analysis of the cell proliferation and biomass accumulation of *E. gracilis* cells. **c** Time course of the changes in the electrical diameters of *E. gracilis* cells at four detection frequencies (500 kHz, 4 MHz, 7 MHz and 10 MHz). **d** Time course of the changes in the electrical opacity of *E. gracilis* cells

Although some inorganic metal ions are required for *E. gracilis* cell growth and are stabilized during biomass synthesis^26,30^, the organic supplies may be insufficient in the natural environment. Thus, *E. gracilis* cells have to grow photoautotrophically, and most of their biomass has to be produced by photosynthesis using carbon dioxide from the air as the carbon source^31,32^. To analyze the effects of inorganic ions on cell growth and biomass accumulation, *E. gracilis* cells were cultured in a 1× PBS solution as a control. The dielectric properties, cell multiplication, and biomass accumulation of the *E. gracilis* cells cultured in PBS and Cramer-Myers (CM) medium are compared and shown in Fig. 5.

Without organic carbon sources in the growth medium, the *E. gracilis* cells cultivated in PBS and CM medium over four days are shown in Fig. 5a. Because of the limited carbon sources in the air, the resulting paramylon synthesis was restricted. Thus, when the *E. gracilis* cells were transferred into CM medium and PBS solution, the cells started to consume stored energy (paramylon), leading to a reduction in biomass. Additionally, the effect of inorganic ions on cell growth is shown in Fig. 5b. Despite insufficient carbon sources, the *E. gracilis* cells in CM medium divided more frequently than those in PBS solution. This result also supported several research conclusions regarding the promotive effects of inorganic ions on *E. gracilis* cell multiplication^33–35^.

Although there were more cells in the CM medium than in the PBS medium, the biomasses of the cells were almost the same in both cases. In other words, the individual cells cultured in CM medium might have less biomass than the cells cultured in PBS solution. This conclusion was further confirmed by impedance detection, as illustrated in Fig. 5c. After four days of cultivation, the electrical diameters of the *E. gracilis* cells in PBS solution were larger than those of the cells in CM medium at four detection frequencies. This indicated that the *E. gracilis* cells in PBS solution had a larger volume due to a larger low-frequency electrical diameter and had denser intracellular components due to a higher electrical opacity (see Fig. 5d) compared to the cells cultured in CM medium.

Additionally, the electrical diameters and electrical opacities of cells are also good indicators of the change in cultivation medium. When the *E. gracilis* cells were transferred to fresh cultivation medium, the electrical diameter and opacity of the cells, especially in PBS solution, declined rapidly on the first day of cultivation, which can be related to the changes in the osmosis and pH value of the cultivation medium. After two days of adaptation to these new environments, the electrical opacity and diameter of the *E. gracilis* cells returned to normal.

Considering the growth conditions of the *E. gracilis* cells in CM medium, KH medium and PBS solution, we concluded that some inorganic ions may contribute to cell multiplication. Especially when *E. gracilis* cells are grown in an environment with sufficient organic and inorganic sources, their proliferation rate and cell biomass productivity reach their maximum values. Inorganic ions can accumulate in the cells^36,37^, and the resultant biomass is valuable as a source of biodiesel.

## Discussion

In this work, we proposed a method employing the distribution of the tilting level of impedance pulses to determine if the detection frequency is sufficient to penetrate cell membranes. At high detection frequencies, the intracellular component distribution could tilt the impedance pulses, which has been verified in simulations and experiments. The experimental results showed that when the electrical penetration of the cell membrane occurs, the distribution of the tilt index is gradually decentralized as the detection frequency increases. Research has found that a detection frequency of 7 MHz is sufficient to penetrate the cell membrane of *E. gracilis* cells.

In living *E. gracilis* cells, there are two types of intracellular components that account for most of the biomass, namely, paramylon and chloroplasts. Paramylon, as a biodiesel source^38,39^, accounts for more than 50% (w/w) of the dry weight of individual *E. gracilis* cells^40^. Another source of biodiesel is the membrane of chloroplasts, which accounts for approximately 22% of the biomass. As an environmentally benign and sustainable alternative, biomass energy has sparked considerable interest within both scientific and industrial communities^4,41^. In Japan, for example, biodiesel made from microalga *Euglena gracilis* (*E. gracilis*) cells has been used in shuttle buses and commercial airplanes as a “next-generation renewable fuel”^42^. Since microalgal cells are heterogenous and their biomass productivity varies with their growth conditions, efficient techniques are needed for monitoring biomass accumulation during cultivation processes. In this work, our study showed that impedance cytometry is applicable for analyzing the cell morphology as well as the intracellular components of *E. gracilis* cells. Additionally, mammalian cells and other types of cells that are important in the fields of biomedicine and with respect to the environment will be analyzed in the future.

For the intracellular components of *E. gracilis* cells, individual paramylon molecules are encased by a biomembrane and often exhibit high degrees of crystallinity, which theoretically contributes to the high current resistance^33,43^. Chloroplasts are double-membrane organelles that result in a higher current resistance at the same detection frequency compared to that of a single membrane cell. For the assessment of intracellular biomass, it is necessary to determine the detection frequency at which the cell interior can be detected. In this work, we reported that 7 MHz is sufficient to penetrate the cell membrane of *E. gracilis* cells. However, the dielectric properties of various organelles and biomolecules are still unknown. According to the simplified numerical model, intracellular components can be polarized at 10 MHz, but in experiments, this frequency is insufficient. Consequently, the dielectric parameters of intracellular components require additional study for a more accurate simulation.

Multifrequency impedance cytometry has been widely employed in single-cell detection and analysis^5,8,44^. In addition, it is well known that high-frequency impedance values are related to intracellular components, as high-frequency currents can propagate through the cell membrane^24^. Since current propagation is not visible in cells, we lack a direct method for determining if the detection frequency is applicable for measuring cell interior components. In this work, we proposed that the distribution of intracellular components can be used to determine the electrical penetration of the cell membrane. Specifically, when the electric penetration of the membrane occurs, the decentralization of the tilt index distribution increases with increasing detection frequency. This is because cell interiors are more heterogeneous than their morphologies in a population. Heterogeneous internal structures would cause the tilt index to become increasingly decentralized.

Herein, we employed low-frequency impedance to conduct a performance analysis of the morphology and volume of single cells and high-frequency impedance to characterize intracellular biomass. The detection mechanism is supported by numerous previous works. Our previous work has shown that low-frequency detection is applicable for analyzing cell morphology, as the low-frequency current mainly propagates around the cell^13,18,19^. The dependence of low-frequency impedance on cell volume has also been verified^8^. Additionally, it has been demonstrated that high-frequency impedance can be used to analyze the amount^19^, distribution^18^, and density^19,45^ of intracellular components. Herein, impedance-based biomass analysis is based the applicability of high-frequency impedance for monitoring the amount and density of intracellular components, which has also been proven by experimental results.

Last, the proposed impedance-based platform has been shown to be applicable for evaluating the effects of the culture conditions on *E. gracilis* cell growth (volume, opacity and number) and biomass accumulation. High-frequency impedance magnitudes (≥7 MHz) can be applied to characterize biomass accumulation, and low-frequency impedance magnitudes (≤4 MHz) allow the quantification of the volume of single cells. The changes in biomass accumulation and cultivation in different media were successfully monitored over four days. In the future, we suggest extending the application of the tilt index to mammalian cells to track changes in membrane properties regarding cell aging, carcinogenesis, or lysis.

## Materials and methods

### Sample preparation

Experiments were performed on *E. gracilis* NIES-48 cells provided by the Microbial Culture Collection at the National Institute for Environmental Studies (NIES, Japan). The cultures were grown in culture tubes each with a working volume of 13 mL under continuous illumination (warm white, 130–150 μmol/m^2^/s) at 28 °C. To study the effects of organic nutrients on the biomass and metabolization of individual cells, *E. gracilis* cells were grown photomixotrophically using KH medium (pH: 3.5)^46^. To study the effects of inorganic ions on cell growth, *E. gracilis* cells were cultivated photoautotrophically using CM medium (pH: 3.9)^47^. *E. gracilis* cells were cultivated using 1× phosphate-buffered saline solution (PBS, pH: 6.9) as the control group. The detailed components of the CM and KH media were described by Wang et al.^35^. Briefly, the CM medium does not include any organic carbon sources, whereas the KH medium contains glucose and various organic acids and amino acids as carbon sources^48^. Both KH and CM media contain high concentrations of inorganic ions, such as Zn^2+^, Mn^2+^, Fe^3+^, Cu^2+^, Co^2+^, and Ni^2+^, some of which can promote the biomass accumulation and multiplication of *E. gracilis* cells^36,49^.

For calibration, 10 μm polystyrene beads (Polysciences, USA) were utilized as reference particles due to their frequency-independent physical properties, which allow them to be considered as perfect insulators. Theoretically, the magnitudes of the four-frequency impedances of beads should be identical^44^.

All samples were transferred to 1×PBS and injected into microfluidic devices using a syringe pump (NIHON KOHDEN CFV-3200). The sample flow rate was 4.5 μL/min, resulting in a throughput of approximately 1250 samples/s for impedance detection in this work. Three replicates of each experiment were generally performed, i.e., in Fig. S3, there were three cell culture groups for each cell culture state.

### Growth detection of E. gracilis

Experiments were conducted over a 4-day period (i.e., 0–4 days) for the CM-medium, KH-medium, and 1×PBS solution, with each group containing three independent cultures of *E. gracilis* for robust characterization. The growth of *E. gracilis* cells was analyzed according to cell number, dry weight, cell volume, and opacity. Herein, the dry weight of *E. gracilis* cells was determined using 0.4 mL of cultures that had been dried at 100 °C for more than 4 h^50^. The volume of the cells was determined using low-frequency electrical diameters ($\left| {Z_{LF}} \right|^{1/3}$), and the volume of intracellular components was determined using high-frequency electrical diameters ($\left| {Z_{HF}} \right|^{1/3}$)^18^. The color changes in the culture tubes over four days are shown in Fig. S3 in the supplementary information, and the green color in the culture tubes becomes darker as the number of cells grows.

### Impedance microfluidic devices

The polydimethylsiloxane (PDMS) microchannel in the detection area is 40 μm wide and 35 μm deep. The channel is placed over two pairs of coplanar electrodes, each pair consisting of one source and one detection electrode (dimensions: 30 μm wide, 30 μm edge-to-edge span, and 80 μm pair span). Each electrode is coated with a 70 nm thick layer of gold (Au) over a 70 nm thick layer of chromium (Cr). The complete fabrication procedure was described in detail in our previous work^13,18^.

### Impedance detection and calibration

As shown in Fig. 1c, a field-programmable gate array (FPGA)-based lock-in amplifier (Diligent Eclypse Z7, USA) was used to generate a 1 V alternative current (AC) signal at four detection frequencies (i.e., 500 kHz, 4 MHz, 7 MHz, and 10 MHz) and perform the real-time processing of the impedance signals^13,20^ from the detection area. The current signals from two detection electrodes were converted into voltage signals with self-developed transimpedance amplifiers (I/V converters) and then compared with differential amplifiers (Diff). The resulting differential voltage was further processed on the FPGA board to obtain the corresponding impedance signals. All impedance signals were recorded at a sampling rate of 62.5 kHz using a data collection device (USB-6363 BNC, National Instruments, USA) and displayed in real time on a computer through NI DAQExpress (National Instruments, USA). The experimental steps for using this device for impedance detection are shown in Fig. S4 in the supplementary information. In Fig. 1d, the frequency response of the I/V converter shows its ability to stably convert current signals of up to 18.48 MHz. When the detection frequency was greater than 10 MHz, the amplification factor (gain) began to decline.

### Processing and characterization of impedance signals

The impedance signals were processed using custom scripts written in MATLAB (version 2021b, MathWorks, USA). The impedance (|Z|) of each bead or *E. gracilis* cell was determined using a single-peak Gaussian fit to extract the peak signal amplitude for each applied frequency. The mean impedance magnitudes of the 10 μm beads at four frequencies were determined automatically and then used to calibrate the electrical diameters of the *E. gracilis* cells. The mean electrical opacity (|*Z*_*HF*_|/|*Z*_500*kH*_|) and diameter ($\left| Z \right|^{1/3}$) of *E. gracilis* cells were normalized using single linear multipliers to ensure that the mean values of both of the impedance parameters of the beads were at opacity = 1 and diameter = 10 at each frequency. Additionally, the morphology and intracellular distribution of the *E. gracilis* cells can be assessed at low and high frequencies, respectively, using the tilt index (*T*^*Left*^⁄*T*^*Right*^ − 1) by comparing the time span of the left and right half (*T*^*Left*^⁄*T*^*Right*^) of the impedance pulses^18^.

## Supplementary information

Supplementary information

Impedance detection of single cells with fluorescence video
