# Supplementary material for: Assessment of the electrical penetration of cell membranes using four-frequency impedance cytometry
Source: Microsyst Nanoeng. 2022 Jun 24;8:68. doi: 10.1038/s41378-022-00405-y (PMC9226050; doi:10.1038/s41378-022-00405-y)
Supplement: Supplementary file 1 — Supplementary information [file 41378_2022_405_MOESM1_ESM.docx]

**Supplementary Information**

**Assessment of electrical penetration of cell membrane using four-frequency impedance cytometry**

Tao Tang^1^, Xun Liu^1^, Yapeng Yuan^2^, Tianlong Zhang^1,3^, Ryota Kiya^1^, Yang Yang^4^, Kengo Suzuki^5^, Yo Tanaka^2^, Ming Li^3^, Yoichiroh Hosokawa^1^ and Yaxiaer Yalikun^1,2^*

1. Division of Materials Science, Nara Institute of Science and Technology, 8916-5 Takayama-cho, Ikoma, Nara 630-0192, Japan.

2. Center for Biosystems Dynamics Research (BDR), RIKEN, 1-3 Yamadaoka, Suita, Osaka, 565-0871, Japan.

3. School of Engineering, Macquarie University, Sydney, 2109, Australia.

4. Institute of Deep-Sea Science and Engineering, Chinese Academy of Sciences, Sanya, Hainan 572000, P.R. China.

5. Euglena Co. Ltd., Tokyo 108-0014, Japan.

* Corresponding authors: Yaxiaer Yalikun, Division of Materials Science, Nara Institute of Science and Technology, Takayama, Ikoma, Nara 630-0192, Japan.

Email: [yaxiaer@ms.naist.jp](mailto:yaxiaer@ms.naist.jp)

# Numerical simulation parameters

Table S1: Simulation parameters

| Parameters | Value |
| --- | --- |
| Channel length | 300 μm |
| Channel depth | 30 μm |
| Relative permittivity of membrane | 3 |
| Relative permittivity of cytoplasm | 50 |
| Conductivity of cytoplasm | 0.5 S/m |
| Electrodes wide | 30 μm |
| Electrode span | 30 μm |
| Relative permittivity of 1× PBS | 80 |
| Conductivity of 1× PBS | 1.34 S/m |

# Tilt index


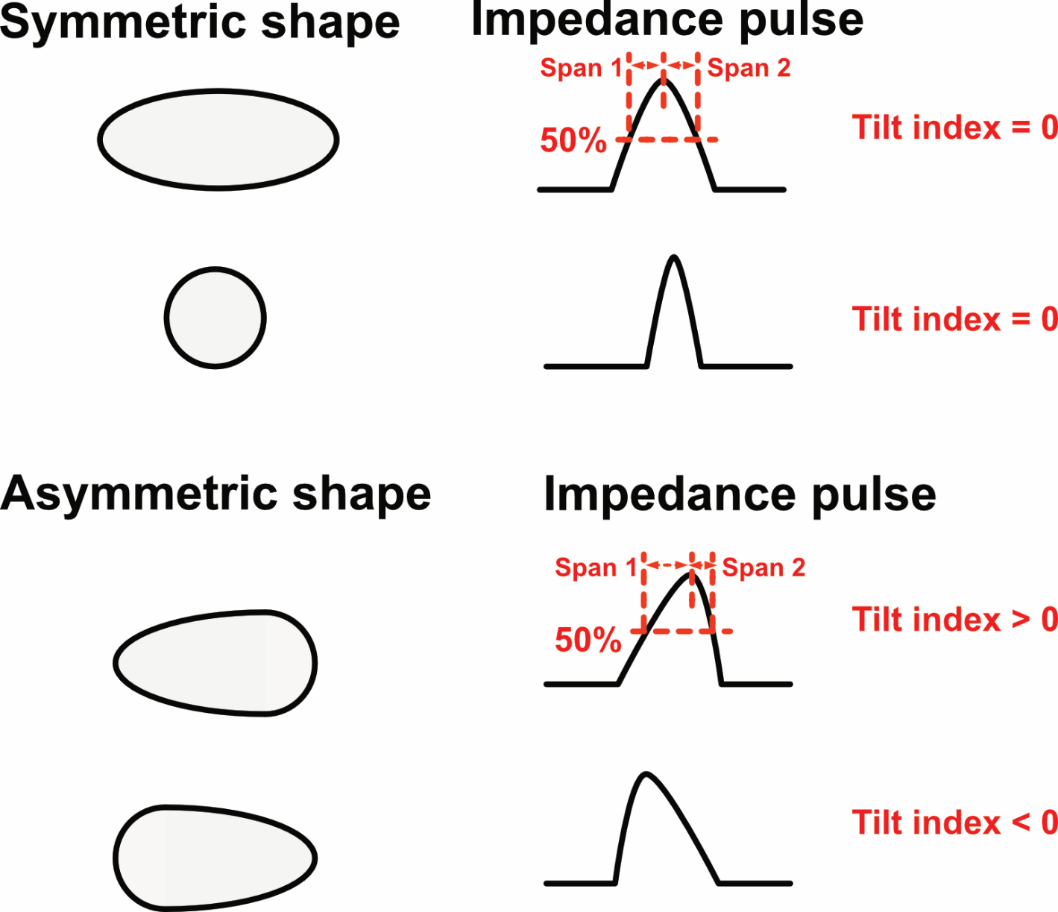


Figure S1 Numerical simulation of tiling impedance pulse analysis for *E. gracilis* cells having different morphologies.

the impedance pulse of spherical beads is not tilted, but the impedance pulse triggered by *E. gracilis* cells is tilted due to the asymmetric shape of *E. gracilis*. To better characterize this phenomenon, we provided a new metric – tilt index, defined as:

$tilt index=\frac{span 1}{span 2}-1$ (1)

The critical line is 50% peak value, and the tilt index is calculated as the ratio of the time spans on either side of the pulse peak and then subtracted by one, so that the metric is independent on the time-related parameters (i.e., flow rate) and the number fluctuates around zero. Theoretically, symmetric beads (e.g., Fig. S3) have a tilt index of almost zero, and the tilt index of most *E. gracilis cells* is greater than zero. More specifically, the tilt index tends to be zero for symmetric objects, while the tilt index tends to be greater than zero for non-symmetric objects. The degree of asymmetry of an object can also be quantified as a specific value by the tilt index. Notably, the sign of tilt index only indicates the orientation of the samples in the detection area.

# Single cell detection


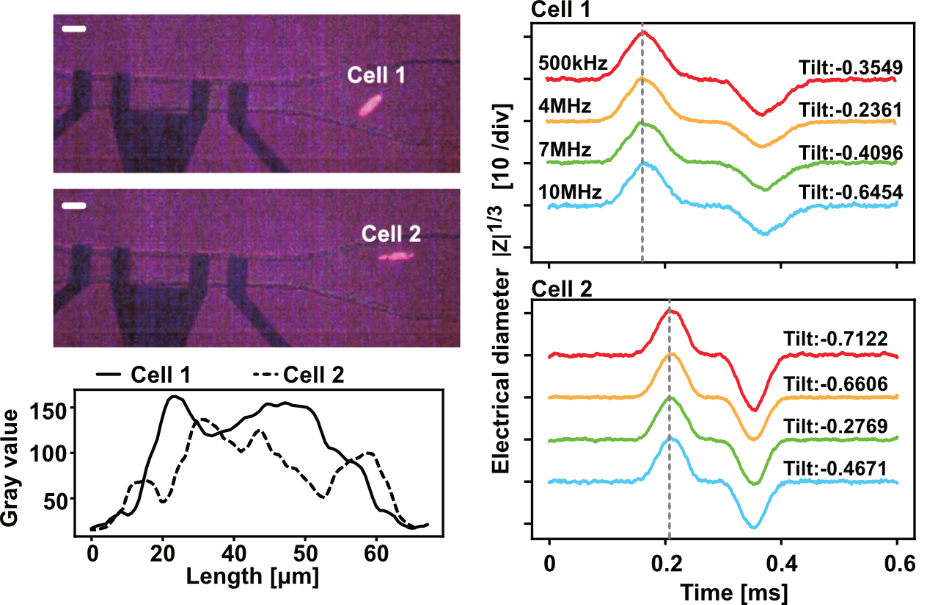


**Figure S2**: Impedance detection of *E. gracilis* cells. Electrical diameter and tilt index of two *E. gracilis* cells with different brightness at four detection frequencies (i.e., 500 kHz, 4 MHz, 7 MHz and 10 MHz). The scale bar indicates 30 μm.

Ultraviolet rays (U-ULH, OLYMPUS OPTICAL CO. LTD.) were employed to stimulate autofluorescence of *E. gracilis* cells to facilitate the impedance detection and visualize intra-cellular component distribution. Through a red-light filter, autofluorescence signals within the 648-709 nm wave-length region were exclusively recorded at a rate of 60 fps using a CCD camera (LU075C-IO, Lumenera Corp.). The suspension of *E. gracilis* cells was injected into the device at a flow rate of around 0.1 μL/min during video recording. Video clip and impedance signals of flowing cells are provided in supplementary information (see Movie S1).

Through the autofluorescence-based detection of the components inside the cell, it can be found that intracellular components were not evenly distributed. Besides, it is also in agreement with the simulation (see Fig. 2(b)), the tilt index induced by individual cells varied with the detection frequency.

# Cell cultivation


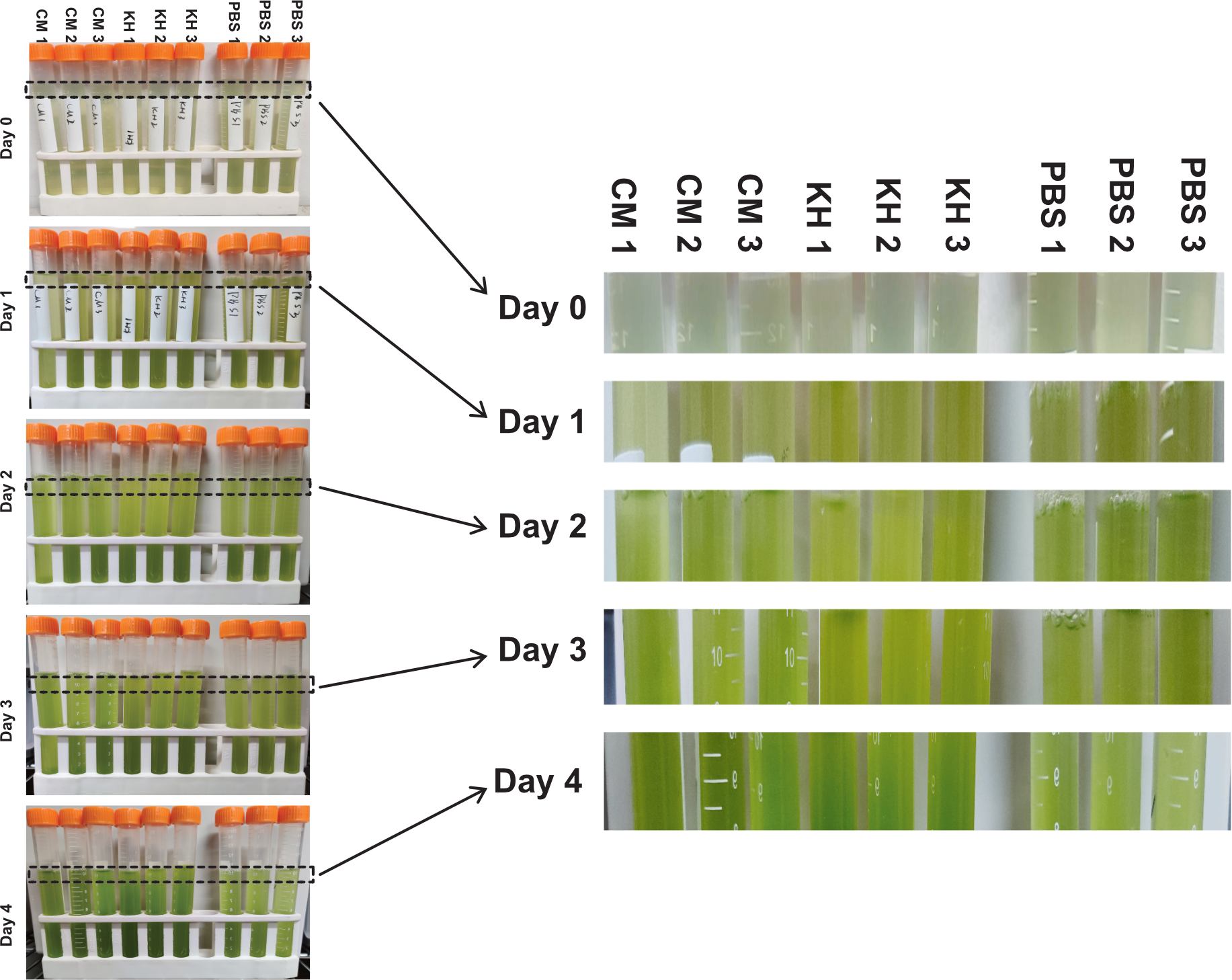


Fig. S3: Color change in culture tubes for *E. gracilis* cells. The green color becomes more intense as more cells multiply each day.

# FPGA-based Impedance detection system


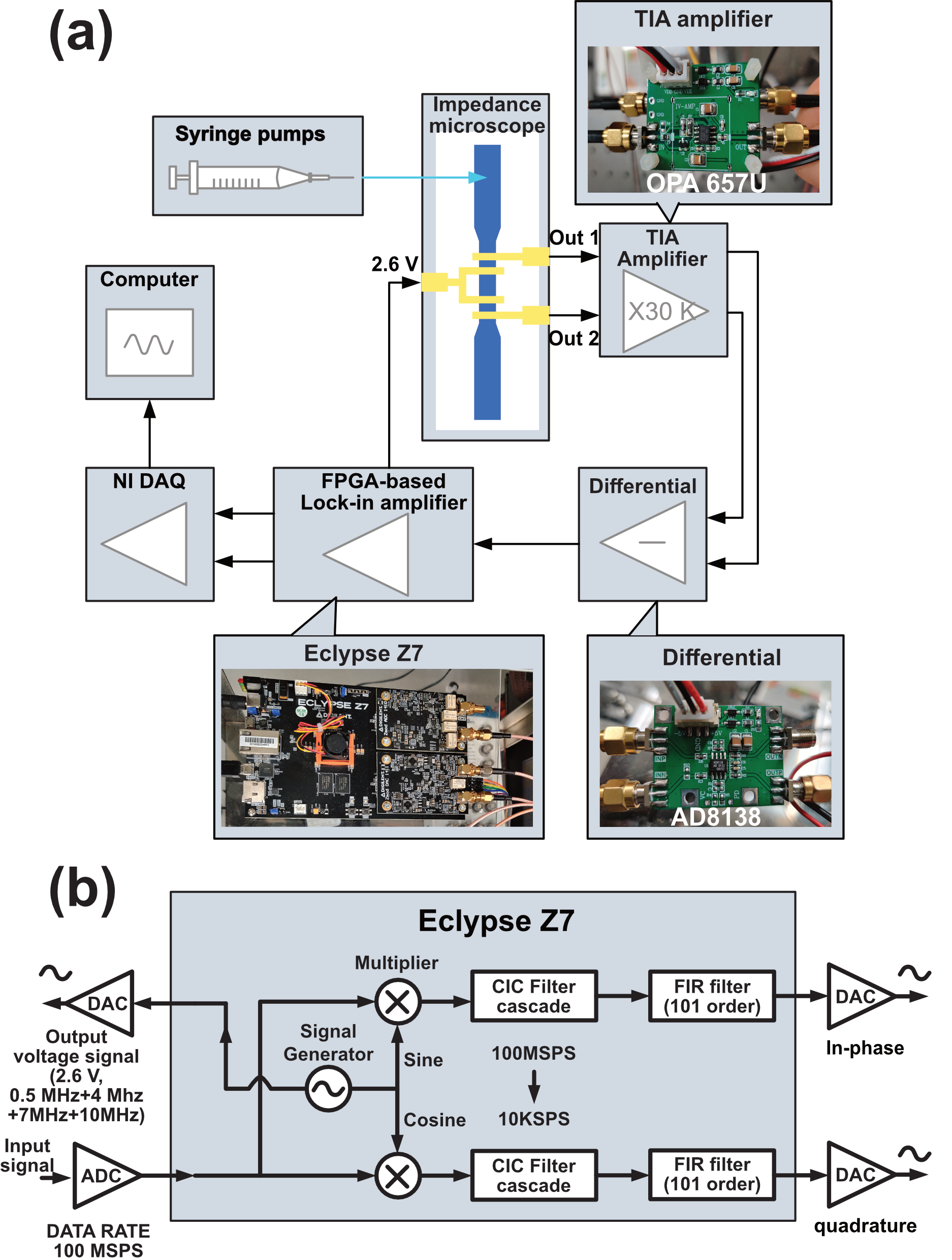


Figure. S4: Experimental setup. (a) Schematic diagram of the detection method, including specific hardware devices. (b) FPGA-based Lock-in amplifier and involved digital signal processing methods.

Fig. S2 (a) illustrates the schematic diagram of impedance cytometry, which consists of a homemade measurement circuit and a FPGA-based LIA. The current signals from two output electrodes, triggered by flowing objects, are converted into voltage signals in Transimpedance Amplifier (TIA). The TIA was made with operational amplifier (OPA 657U), and the feedback resistor was selected as 30 kΩ for the best performance in this case. Then, the voltage signals from the TIA were sent into a differential amplifier (AD 8138) to calculate the differential voltage signals between two output electrodes. The fluctuation in differential voltage can be detected by the input side of the FPGA-based LIA through analog-to-digital converter (ADC) with a sampling frequency of 100 mega samples per second (MSPS), which is more than 10 times higher than the frequency of target voltage signals (500 kHz, 4 MHz, 7MHz, 10 MHz).

In this work, we applied (Xilinx ZYNQ7020) to prepare the LIA. Fig. S2(b) depicts the detection algorithms. The codes were compiled using VIVADO 2021.1. Inside the FPGA platform, a digital signal generator generates the reference signals (i.e., the in-phase and quadrature signals), one of which is given as the excitation voltage (2.6 V peak-to-peak) for the impedance cytometry. In the experiment, the detection frequency was set to 500 kHz, 4MHz, 7Mhz, and 10 MHz, and the frequency is equal to the frequency of the signals to be measured from the output sides. Then, the measured voltage signals were modulated with the in-phase and quadrature signals via digital multipliers in real time, resulting in two components (i.e., DC components and high-frequency components). After that, both results were sent to the next stage – the cascaded integrator–comb (CIC) filter cascade, in which the sampling frequency was reduced to 10 KSPS to eliminate most high-frequency components. To further reduce noise, we used a finite impulse response (FIR) filter with an order of 101 (Peled and Bede Liu, 1974). The filter design was achieved using MATLAB 2021b (Mathworks Inc., Natick, USA). The outputs from the FIR filter were converted to analog signals again (DIGILENT Pmod DA2, National Instruments), recorded by the data collection system (USB-6363 BNC, National Instruments, USA) and presented in computer.
